# No evidence of an association of multiple sclerosis (MS) with Borna disease virus 1 (BoDV-1) infections in patients within an endemic region: a retrospective pilot study

**DOI:** 10.1007/s15010-023-02099-4

**Published:** 2023-10-09

**Authors:** Markus Bauswein, Gertrud Knoll, Barbara Schmidt, André Gessner, Bernhard Hemmer, Martina Flaskamp

**Affiliations:** 1 Institute of Clinical Microbiology and Hygiene, University Hospital Regensburg, Regensburg, Germany; 2 Department of Neurology, School of Medicine, Technical University of Munich, Klinikum rechts der Isar, Munich, Germany; 3 Munich Cluster for Systems Neurology (SyNergy), Munich, Germany

**Keywords:** Borna disease virus (BoDV-1), Encephalitis, Multiple sclerosis (MS), ELISA, iIFA, Serology

## Abstract

**Background:**

Borna disease virus 1 (BoDV-1) causes rare human infections within endemic regions in southern and eastern Germany. The infections reported to date have been linked to severe courses of encephalitis with high mortality and mostly irreversible symptoms. Whether BoDV-1 could act as a trigger for other neurological conditions, is, however, incompletely understood.

**Objectives and methods:**

In this study, we addressed the question of whether the presentation of a clinically isolated syndrome (CIS) or of multiple sclerosis (MS) might be associated with a milder course of BoDV-1 infections. Serum samples of 100 patients with CIS or MS diagnosed at a tertiary neurological care center within an endemic region in southern Germany and of 50 control patients suffering from headache were retrospectively tested for BoDV-1 infections.

**Results:**

In none of the tested sera, confirmed positive results of anti-BoDV-1-IgG antibodies were retrieved. Our results support the conclusion that human BoDV-1 infections primarily lead to severe encephalitis with high mortality.

## Introduction

The hypothesis of a viral infection triggering demyelination has long been discussed as possible pathomechanism contributing to multiple sclerosis (MS). A recently published study reported a 32-fold increased risk of acquiring MS after infection with Epstein-Barr virus (EBV), but not after infection with human cytomegalovirus (CMV) [3]. In addition, the treatment with recombinant human interferon (IFN-)β, which has both immunomodulatory and antiviral properties, is a well-established disease-modifying therapy for clinically isolated syndrome (CIS) and relapsing MS. A recent epidemiological study screened patients with encephalitis and different neuropsychiatric conditions in Germany for infections with Borna disease virus 1 (BoDV-1) [1], a highly neurotropic virus for which rare spill-over infections from shrews to humans leading to severe courses of encephalitis with mostly fatal outcome have recently been described within endemic regions in southern and eastern Germany [5, 7, 8, 10, 11, 13, 15]. As patients diagnosed with CIS or MS were not included in this analysis, we conducted a complementary study addressing the question of whether a clinically isolated syndrome (CIS) or relapsing–remitting MS (RRMS) with fulfilled McDonald criteria might be associated with a milder course of Borna disease virus 1 (BoDV-1) infections.

## Methods

### Study cohorts

Stored serum samples of 100 patients with CIS (*N* = 25) and RRMS (*N* = 75) of a tertiary neurological care center within a BoDV-1-endemic region in southern Germany were included in the study. Patients were selected from a larger cohort based on the diagnosis of a CIS or a RRMS, either stable (30%) or unstable (70%) under current or previous therapy with IFN-β. The ratio of CIS to RRMS patients in the selected group resembled the one in the larger cohort. As a control cohort, serum samples of patients suffering from headaches without a structural correlate (*N* = 50), recruited at the same center, were used. The retrospective analysis of stored patients´ samples was approved by the Ethics Committee of the Technical University of Munich (reference number 160/20 S). Residence of > 90% of patients was in the German state of Bavaria for which most of human BoDV-1 infections have been reported so far. Further characteristics of included patients are shown in Table 1.

**Table 1:** Characteristics of study cohorts

	CIS/MS patients (*N* = 100)	Control patients (*N* = 50)
Age (years), median [IQR]	34 [27–40.75]	36.5 [30.5–46.25]
Female gender	*N* = 71 (71%)	*N* = 34 (68%)
Diagnosis	Clinically isolated syndrome (CIS): *N* = 25 (25%) Relapsing remitting multiple sclerosis (RRMS): *N* = 75 (75%)	Headaches without structural correlate: *N* = 50 (100%)
Disease duration (days) at blood sampling, median [IQR]	582 [21–2372]	
Disease modifying therapy (DMT) at blood sampling:		
No DMT	*N* = 44 (44%)	
Interferon β	*N* = 28 (28%)	
Natalizumab	*N* = 17 (17%)	
Fingolimod	*N* = 5 (5%)	
Dimethyl fumarate	*N* = 2 (2%)	
Ocrelizumab	*N* = 2 (2%)	
Glatiramer acetate	*N* = 1 (1%)	
Daclizumab	*N* = 1 (1%)	

### BoDV-1 ELISA

As a serological screening assay, a recently published BoDV-1 ELISA system using recombinantly expressed viral N, X and P proteins as antigens was performed [10]. In brief, serum samples were pre-diluted 1:100. As secondary antibody, a horseradish-peroxidase (HRP)-conjugated polyclonal rabbit anti-human-IgG antibody (Agilent, Santa Clara, CA, USA) was used in a 1:5,000 dilution. 50 µL of TMB (Mikrogen, Neuried, Germany) served as substrate. Reaction was stopped after 4 min of incubation at room temperature (RT) with Mikrogen Stopp Solution (containing 24.9% H_3_PO_4_). Optical density was determined at 450 nm and 630 nm (background). To control for inter-assay variability, all sample values were normalized to an external standard sample. Sample-to-cut-off (S/CO) values were calculated using previously established cut-offs for each antigen. With the exception of 12 sera of the control cohort, which were tested negative, each ELISA was carried out twice to four-times for all serum samples in order to control for assay precision. Medians of S/CO values were used for further analysis.

### BoDV-1 indirect immunofluorescence assay (iIFA)

Samples with reactions against at least one ELISA antigen were re-tested by a BoDV-1 indirect immunofluorescence assay (iIFA), which was performed as previously described [10, 13]. For the iIFA, a Vero cell culture persistently infected with a BoDV-1 isolate derived from human brain tissue (isolate Regensburg 2019) was used [11]. Infected Vero cells were mixed 1:2 with uninfected Vero cells and cultured in 96-well microtiter plates (Ibidi, Gräfelfing, Germany) overnight to achieve confluent cell layers. As negative controls, uninfected Vero cells were prepared in the same manner. The next day, supernatants were removed, plates were dried for 2 h and fixed at 80 °C for 2 h. Prepared plates were stored at − 20 °C before use. Heat-inactivated patient samples were added in a twofold dilution series (1:20, 1:40, 1:80). As secondary antibody, a Cy-3-conjugated polyclonal rabbit anti-human-IgG antibody (Jackson ImmunoResearch, West Grove, PA, USA) was used in a 1:200 dilution. Samples were analyzed by fluorescence microscopy. Samples with characteristic fluorescing spots in the nuclei of BoDV-1-infected Vero cells were considered positive. When similar fluorescence signals were detected in non-infected and BoDV-1-infected Vero cells, signals were considered unspecific.

### Data analysis

The data were analyzed by GraphPad Prism, version 9.4.1 (GraphPad Software, San Diego, CA, USA). For contingency analysis, Fisher´s exact test was performed as indicated. Figures were created using GraphPad Prism.

## Results

Serum samples of all patients diagnosed with CIS (*N* = 25) or RRMS (*N* = 75) as well as all samples of control patients suffering from headaches without a structural correlate (*N* = 50) were screened by a recently published in-house BoDV-1 ELISA [10]. Samples with the reactivity against at least one of the recombinant BoDV-1 antigens were re-tested using an indirect immunofluorescence assay (iIFA) as described previously [10, 13].

**Fig. 1:**
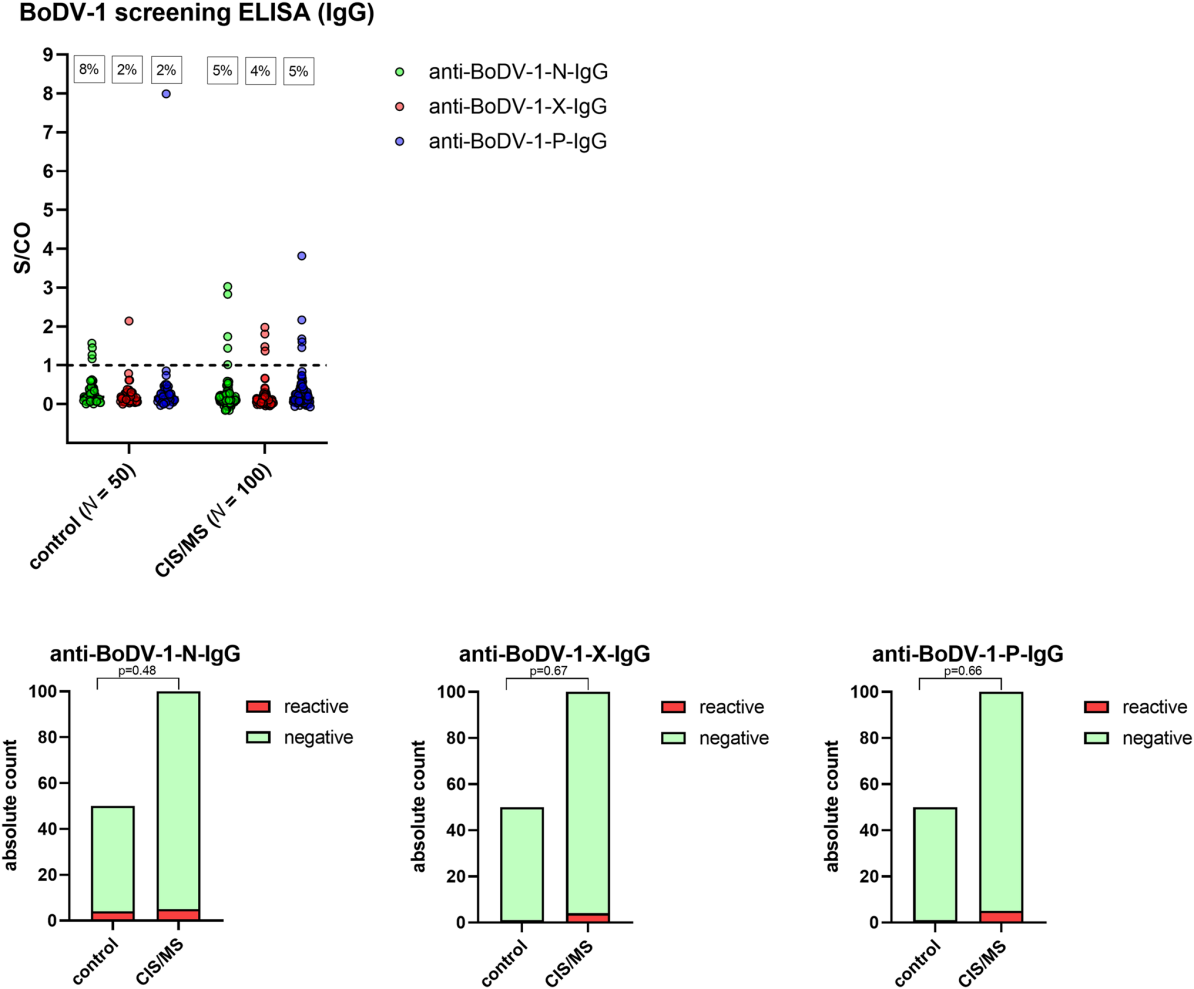
BoDV-1 screening ELISA. Serum samples of patients with headache (control, *N* = 50) and with clinically isolated syndrome (CIS)/multiple sclerosis (MS) (*N* = 100) were screened by a BoDV-1 IgG ELISA system using recombinant viral nucleocapsid (N), X or phosphoprotein (P). With the exception of 12 sera of the control cohort, which were tested negative, the ELISA was carried out twice to four-times for all serum samples in order to control for assay precision. Medians of sample-to-cut-off (S/CO) values were used for further analysis. All ELISA-reactive samples (S/CO > 1) were confirmed to be negative by an indirect immunofluorescence assay (iIFA). **A** shows medians of individual S/CO values. The dotted line represents the ELISA cut-off. Percentages of reactive samples are given. Under **B** absolute counts of reactive and negative samples are shown. Control and CIS/MS cohort were not significantly different based on Fisher´s exact test

ELISA reactivity rates within the cohort of patients with CIS and RRMS were 5% (*n* = 5) for anti-BoDV-1-N-IgG, 4% (*n* = 4) for anti-BoDV-1-X-IgG and 5% (*n* = 5) for anti-BoDV-1-P-IgG (Fig. 1A). A total of 2% (*n* = 2) of samples were reactive against two recombinant ELISA BoDV-1 antigens, while none of the sera was tested reactive against all three ELISA antigens.

For the subgroup of patients with CIS (*N* = 25), five samples (20%) were reactive against a single ELISA antigen (BoDV-1-N-IgG: 1; BoDV-1-X-IgG: 2; BoDV-1-P-IgG: 2), one sample (4%) was reactive against two antigens (BoDV-1-X + BoDV-1-P), while for the subgroup of patients with RRMS reactivity rate against a single antigen was 7% (*n* = 5) (BoDV-1-N: 3; BoDV-1-P: 2) and against two antigens was 1% (*n* = 1) (BoDV-1-N + BoDV-1 P). Based on Fisher´s exact test, reactivity rates against at least one ELISA BoDV-1 antigen were not statistically different for both subgroups (*p* = 0.07).

All ELISA-reactive serum samples were, however, tested negative in the confirmatory BoDV-1 iIFA (detecting IgG antibodies). Thus, all samples were interpreted to be negative for specific anti-BoDV-1-IgG antibodies and BoDV-1 ELISA results were interpreted as false-reactive. Rates of false reactivity of the ELISA system within the CIS/RRMS cohort were not statistically different from the ones within the control cohort (anti-BoDV-1-N-IgG: *n* = 4 (8%), *p* = 0.48; anti-BoDV-1-X-IgG: *n* = 1 (2%), *p* = 0.67; anti-BoDV-1-P-IgG: *n* = 1 (2%), *p* = 0.66) based on Fisher´s exact test (Fig. 1B) and were similar to those of a previously published cohort of healthy blood donors [2]. In summary, no association of CIS or RRMS with confirmed BoDV-1 infections was found in the tested study cohort.

## Discussion

The zoonotic potential of BoDV-1 was proven in 2018 [8, 13]. Since then, approx. 50 human infections have been reported to German health authorities [12]. Evidence is growing that human BoDV-1 infections primarily lead to severe encephalitis with high case fatality [2, 14]. A recent epidemiological study did not find an association with neuropsychiatric disorders [1]. Our result adds the notion that CIS or MS is not regularly associated with high titers of bornavirus-reactive IgG antibodies in serum. Of diagnostic interest, patients with CIS tended to show higher rates of false reactivity in the screening ELISA (effect however not statistically different) with unconfirmed results in the confirmatory iIFA, underlining a possible diagnostic challenge with cross-reactivity in assays based on recombinant BoDV-1 antigens in the acute phase of immunological diseases. After a prodromal phase, most of the human BoDV-1 infections reported so far lead to severe and in most cases irreversible neurological symptoms such as ataxia, paresis, memory loss, seizures and eventually deep coma. Initially, some cases mimicked the clinical presentation of a Guillain-Barré or Miller-Fisher syndrome [2, 4]. The diagnosis is sometimes challenging, as neither normal white blood count (WBC) in cerebrospinal fluid (CSF) nor an unremarkable cMRI definitively rule out the infection at an early stage [6, 9]. In addition, the sensitivity of RT-qPCR from CSF is limited due to the tight cell association of the virus [1]. Thus, RT-qPCR and serological assays should always be performed in parallel. As the disease is rare, awareness must be raised especially in endemic areas in southern and eastern Germany where infected reservoir animals (*Crocidura leucodon*) or sentinel spill-over hosts such as horses and alpacas are reported.

## Limitations

As the study was designed as a pilot project and thus the number of patients included was limited, further research may be warranted to definitely rule out a statistical association of MS and BoDV-1 infections. In addition, low titers of antibodies may not be detectable by the used method, as a 1:100 dilution was used for the screening ELISA.

## Data Availability

The datasets generated in this retrospective analysis are available from the corresponding author M.B. on reasonable request.
